# Transcriptional analysis of liver from chickens with fast (meat bird), moderate (F1 layer x meat bird cross) and low (layer bird) growth potential

**DOI:** 10.1186/s12864-018-4723-9

**Published:** 2018-05-02

**Authors:** Nicky-Lee Willson, Rebecca E. A. Forder, Rick Tearle, John L. Williams, Robert J. Hughes, Greg S. Nattrass, Philip I. Hynd

**Affiliations:** 1 School of Animal and Veterinary Sciences, The University of Adelaide, Roseworthy, SA 5371 Australia; 2 Poultry CRC, University of New England, PO Box U242, Armidale, NSW 2351 Australia; 3 Davies Research Centre, School of Animal and Veterinary Sciences, The University of Adelaide, Roseworthy, SA 5371 Australia; 4 South Australian Research and Development Institute (SARDI), Pig and Poultry Production Institute, Roseworthy, SA 5371 Australia; 5 South Australian Research and Development Institute (SARDI), Livestock and Farming Systems, Roseworthy, SA 5371 Australia; 6 Institute for Future Farming Systems, Central Queensland University, Rockhampton, QLD 4702 Australia

**Keywords:** RNS-seq, Meat bird, Layer, Liver, Functional analysis, FoxO

## Abstract

**Background:**

Divergent selection for meat and egg production in poultry has resulted in strains of birds differing widely in traits related to these products. Modern strains of meat birds can reach live weights of 2 kg in 35 d, while layer strains are now capable of producing more than 300 eggs per annum but grow slowly. In this study, RNA-Seq was used to investigate hepatic gene expression between three groups of birds with large differences in growth potential; meat bird, layer strain as well as an F1 layer x meat bird. The objective was to identify differentially expressed (DE) genes between all three strains to elucidate biological factors underpinning variations in growth performance.

**Results:**

RNA-Seq analysis was carried out on total RNA extracted from the liver of meat bird (*n* = 6), F1 layer x meat bird cross (*n* = 6) and layer strain (*n* = 6), males. Differential expression of genes were considered significant at *P* < 0.05, and a false discovery rate of < 0.05, with any fold change considered. In total, 6278 genes were found to be DE with 5832 DE between meat birds and layers (19%), 2935 DE between meat birds and the cross (9.6%) and 493 DE between the cross and layers (1.6%). Comparisons between the three groups identified 155 significant DE genes. Gene ontology (GO) enrichment and Kyoto Encyclopaedia of Genes and Genomes (KEGG) pathway analysis of the 155 DE genes showed the FoxO signalling pathway was most enriched (*P* = 0.001), including genes related to cell cycle regulation and insulin signalling. Significant GO terms included ‘positive regulation of glucose import’ and ‘cellular response to oxidative stress’, which is also consistent with FoxOs regulation of glucose metabolism. There were high correlations between FoxO pathway genes and bodyweight, as well as genes related to glycolysis and bodyweight.

**Conclusions:**

This study revealed large transcriptome differences between meat and layer birds. There was significant evidence implicating the FoxO signalling pathway (via cell cycle regulation and altered metabolism) as an active driver of growth variations in chicken. Functional analysis of the FoxO genes is required to understand how they regulate growth and egg production.

## Background

Advancement in livestock production through selective breeding is perhaps best demonstrated in the poultry industry, where genetic selection, combined with advances in nutrition and improved management, have resulted in increases in meat bird growth in excess of 400% over the past 50 years [1]. Despite intense selection, there is still a significant amount of performance variation observed in commercial meat bird flocks, for both growth and feed efficiency [2, 3]. Feed costs account for ~ 70% of the variable costs of production in chicken meat enterprises [4], therefore optimizing performance is of economic importance to the producer and industry alike. Increased growth has not been achieved without unfavourable consequences as modern meat strains now predisposed to; excessive fat deposition [5, 6], increased leg deformities/lameness [7], metabolic disorders including; pulmonary hypertension, ascites, and sudden death syndrome [8, 9], and altered immune function [10], particularly when compared to slower growing lines such as layers and heritage line meat birds used in these studies.

Studies of different lines of chicken have explored physiological and/or anatomical growth constraints due to differential selection pressure. For example, comparison of heritage line meat birds unselected for growth, and commercial meat birds, demonstrates gross increases in breast muscle mass in modern meat birds [11]. A major difference was identified at d 14 post hatch where breast muscle growth of the heritage line plateaued at ~ 9% of total bodyweight, while breast muscle continued to increase in the commercial strain (at 14% of total bodyweight at d 14 to ~ 18% by d 28). Conversely, organs such as the heart, lungs and digestive system [1, 11, 12] have been shown to decrease as a percentage of bodyweight compared to heritage strains.

Similarly, comparative studies of strains allow for identification of physiological constraints. Experimental models of meat birds identified differential fatty acid metabolism in birds selected for either high or low abdominal fat [13, 14], or very low density lipoprotein (VLDL) plasma concentrations [15]. Comparisons of these lines, regardless of nutritional status, shows that total plasma lipids and lipoprotein levels are higher in the fat line, suggesting a higher rate of hepatic lipogenesis in fat-line birds [16]. Transcriptional analyses of genetically lean and fat chickens [17, 18] as well as juvenile and mature laying hens [19] also reveals differential expression (DE) and regulation of lipogenic genes. Additionally, fat-line birds have been shown to have significant activation of the early steps of insulin signalling at 9 wks post hatch, which may partially account for the increased lipogenesis in the liver [20]. Comparisons of domestic meat birds with the ancestral red jungle fowl, identified an intestinal glucose uptake ‘surge’ by means of increased brush border glucose transporter activity in meat birds at 2 wks post hatch, not seen in the red jungle fowl [21]. The general finding was that the meat birds had decreased glucose transporter activity (with the exception of wk. 2), but had higher glucose transporter capacity, due to an overall increase in small intestinal mass. Furthermore, modern meat birds have been shown to be less immunologically responsive to immune challenges in comparison to heritage lines [10] and more recent studies have associated gut microbes with improved feed conversion ratio (FCR; feed intake per unit of bodyweight gain) in meat birds [22, 23]. These examples are far from exhaustive, but highlight the value of comparing phenotypically different breeds and/or lines with different trait selection histories, to identify key biological pathways involved.

Meat and layer strain chickens have undergone differential genetic selection, with meat strains for high carcass yield and feed efficiency (reduced feed conversion ratio), and layers for high egg production and also reduced feed conversion ratio [24], but also lower bodyweight. Selection pressure on different traits has resulted in meat and layer stains with vastly divergent growth potential, with the bodyweights of meat birds being five times that of layers by d 42 post hatch [25]. This divergent growth rate makes meat birds and layers an excellent phenotypic model to study the underlying biological mechanisms contributing to growth and performance (i.e. FCR). However, negative consequences associated with rapid growth rates of meat birds can complicate comparisons, particularly metabolic disturbances, which in themselves may be associated with dramatic shifts in gene expression. In order to bridge the phenotypic gap between meat birds and layers, we used an intermediate growth phenotype for comparison by crossing layer ISA Brown roosters with a line of commercial meat bird breeder hens, producing an F1 layer x meat bird cross.

RNA-Sequencing (RNA-Seq) has recently been used to explore gene expression in livers of juvenile and laying hens to assess differences in the transcriptome at the different developmental stages [19] and also to study differences in the transcriptome of abdominal fat between genetically lean and fat strains of meat birds [18]. In the current study, we hypothesised that genes driving growth and performance variation in poultry could be discovered in genes DE between three groups of birds with differing growth potentials. We utilised our previous differential growth phenotypes [26] to compare the liver transcriptomes of meat birds, F1 layer x meat bird crosses and layer line males at d 14 post hatch. Day 14 was selected as the primary sampling time point due to the rapid increase in growth observed in meat strains, of which the aim was to capture transcriptional changes at the beginning of this phase for comparison with slower growing birds. The objective was to identify genes and biological pathways contributing to growth and performance differences.

## Methods

### Birds and management

The University of Adelaide Animal Ethics committee (approval #S-2015-171) and the PIRSA Animal Ethics committee (approval # 24/15) approved all procedures. In total, 150 newly hatched male chicks were obtained from the HiChick Breeding Company Pty Ltd., Bethel, South Australia; *n =* 50 meat birds (commercial line), *n* = 50 F1 layer (Isa Brown cockerels) x meat bird (commercial line) crosses and *n* = 50 layers (Isa Brown). Chicks were placed in a 6 unit rearing pen (*n* = 25 birds/pen), separated in breed groups (*n* = 2 pens/breed) in a temperature controlled room at the SARDI PPPI Poultry Research Unit, Roseworthy Campus, The University of Adelaide. All birds were fed a standard commercial meat bird starter diet *ad libitum* with no added in-feed antimicrobials or coccidiostats, and had unrestricted access to water via nipple drinker lines. The three experimental groups of males were chosen for their growth potential: fast growing (meat bird), moderate (F1 layer x meat bird) and slow growing (layer strain). Feed conversion ratios were recorded weekly as was bodyweight and bodyweight gain. On d 14 post hatch, 36 birds (*n =* 12 birds/breed) were randomly selected and euthanised by cervical dislocation. Liver tissue samples were rapidly collected, snap-frozen in liquid nitrogen and stored at − 80 °C for RNA extraction and RNA-sequencing.

### RNA extraction

Samples were randomly selected for total RNA extraction (*n* = 6/strain) using an RNeasy Plus Mini Kit (Qiagen, Hilden, Germany). Approximately 80 mg of frozen (− 80 °C) liver tissue was homogenised in 2 mL of Trizol reagent (Invitrogen, Carlsbad, CA). 1 mL aliquots of the Trizol homogenate were combined with 200 μL of chloroform and centrifuged for 15 mins at 4 °C. The upper aqueous phase (350 μL) was transferred to a gDNA eliminator spin column and centrifuged at > 8000 *g* (14,000 rpm) for 30 s. The flow through (300 μL) was collected and mixed with an equal volume of 70% ethanol and transferred onto RNeasy columns. The remaining collection and wash steps were performed according to the manufacturer’s instructions. RNA was eluted in 200 μL of RNA-free water. Purity and concentration was determined using UV spectrophotometry (Nanodrop 1000; Thermo Scienfic, Wilmington, DE).

### RNA-Seq library construction and sequencing

RNA-Seq was carried out by the ACRF Cancer Genomics Facility, Adelaide, SA. The sample quality was analysed on an Agilent Bio-analyser (minimum RIN requirement of 7) and sequencing libraries were made using 2 μL of total RNA. PolyA mRNA isolation was performed using oligo dT beads. Libraries were prepared using KAPA Library Quantification Kits for Illumina platforms (KAPABiosystems, Massachusetts, USA). 2 × 100 nt sequencing was carried out on an Illumin HiSeq 2500 Sequencing System to generate a minimum depth of 25 million reads.

### RNA-Seq analysis

Reads were returned in fastq format. FastQC and adaptor sequences were trimmed from the 3′ end of reads with Cutadapt [27]. Hisat2 [28] was used to map reads to the reference genome Galgal5.0 (ftp://ftp.ncbi.nlm.nih.gov/genomes/Gallus_gallus). Duplicate reads were then removed. Stringtie [28] was used to define the transcripts from the read mappings for each sample, and to merge the transcript definitions for all samples. Transcripts were cleaned up using in-house scripts. The number of raw read counts were calculated for each transcript and sample using the function feature Counts of the R package Rsubread [29]. Another R package, edgeR [30] was used to analyse differential gene expression using normalised counts per million transcripts (CPM) to correct for varying depth of sequence among samples. Transcript data were aggregated by gene. Genes where the maximum CPM was < 1 were removed. Gross transcriptome relationships between the three types of bird were analysed by multidimensional scaling of the CPMs.

### Functional annotation analysis and statistical analysis

Functional enrichment of the DE genes between meat bird vs layer, meat bird vs cross and layer vs cross and DE between all three groups was conducted for gene ontology (GO) terms and Kyoto Encyclopedia of Genes and Genomes (KEGG) pathways using the web based tools in DAVID [31, 32]. Only GO terms and KEGG pathways with *P* < 0.05 were taken into account as significantly enriched among the DE genes. Phenotypic data, including bodyweight, bodyweight gain and liver weights (normalised and actual), were analysed by a one-way ANOVA using SPSS (IBM SPSS Statistics 22). Gene expression levels were correlated with individual bodyweights (all three groups combined) using a Pearson’s correlation in SPSS (IBM SPSS Statistics 22).

## Results

### Phenotypic data

**Fig. 1:**
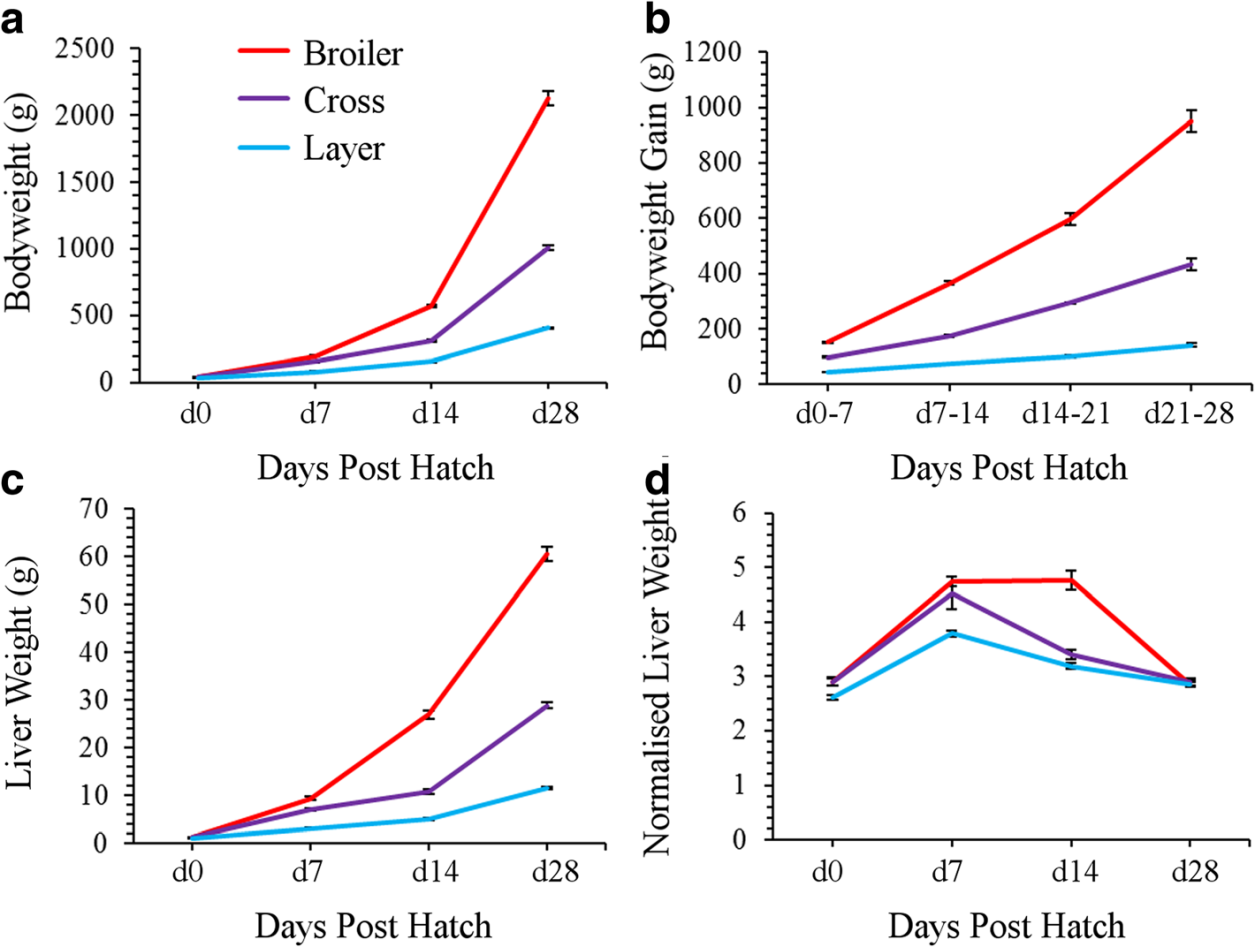
Growth of liver in meat bird, cross and layer strains; **a** Bodyweight (g) vs days post hatch; **b** Bodyweight gain (g) vs weekly intervals; **c** Normalised liver weight (liver weight/total bodyweight) × 100 vs days post hatch; **d** Liver weight vs days post hatch. Error bars are ± SEM

Bodyweight, bodyweight gain, and liver phenotypic data are presented in Fig. 1. Starting bodyweights (mean ± SEM) at hatch were significantly different between meat bird (44.4 ± 0.4 g); cross (42.5 ± .04 g; *P* = 0.008) and layer birds (38.5 ± 0.4 g; *P* < 0.001). At d 14 post hatch, the time of RNA-Seq analysis, bodyweight was significantly different (*P* < 0.001) between all three groups; meat bird (560 ± 8 g); cross (311 ± 8 g) and layer birds (159 ± 2 g). Bodyweight remained different (*P* < 0.001) between the three groups for the remainder of the growth period to d 28, with final bodyweights (mean ± SEM) for meat birds (2102 ± 35 g); cross (1037 ± 31 g) and layers (403 ± 6 g).

Day 0 liver weights (mean ± SEM) did not differ between the meat birds (1.30 ± 0.04 g) and cross (1.19 ± 0.04 g; Fig. 1c). The layer livers (0.99 ± 0.02 g) were however significantly lighter than the meat bird (*P* < 0.001) and cross (*P* = 0.002) livers. From d 7 onwards, liver weights were significantly different (at *P* < 0.001) between all three groups for d 7,-14 and − 28. Normalised liver weights (liver weight/ bodyweight × 100; Fig. 1d) reached maximum weight in layers and crossed birds at d 7 post hatch and declined thereafter. Meat birds had a higher relative ratio and reached maximum relative liver weight later at d 14 post hatch, which was significantly different from cross (*P* < 0.001) and layer birds (*P* < 0.001). The meat birds had a more pronounced decline in relative liver weight compared to cross and layer birds between d 14-d 28. By d 28, there was no difference in normalised liver weight between any of the groups (*P >* 0.05).

### Identification of expressed transcripts and gross transcriptional relationships

**Fig. 2:**
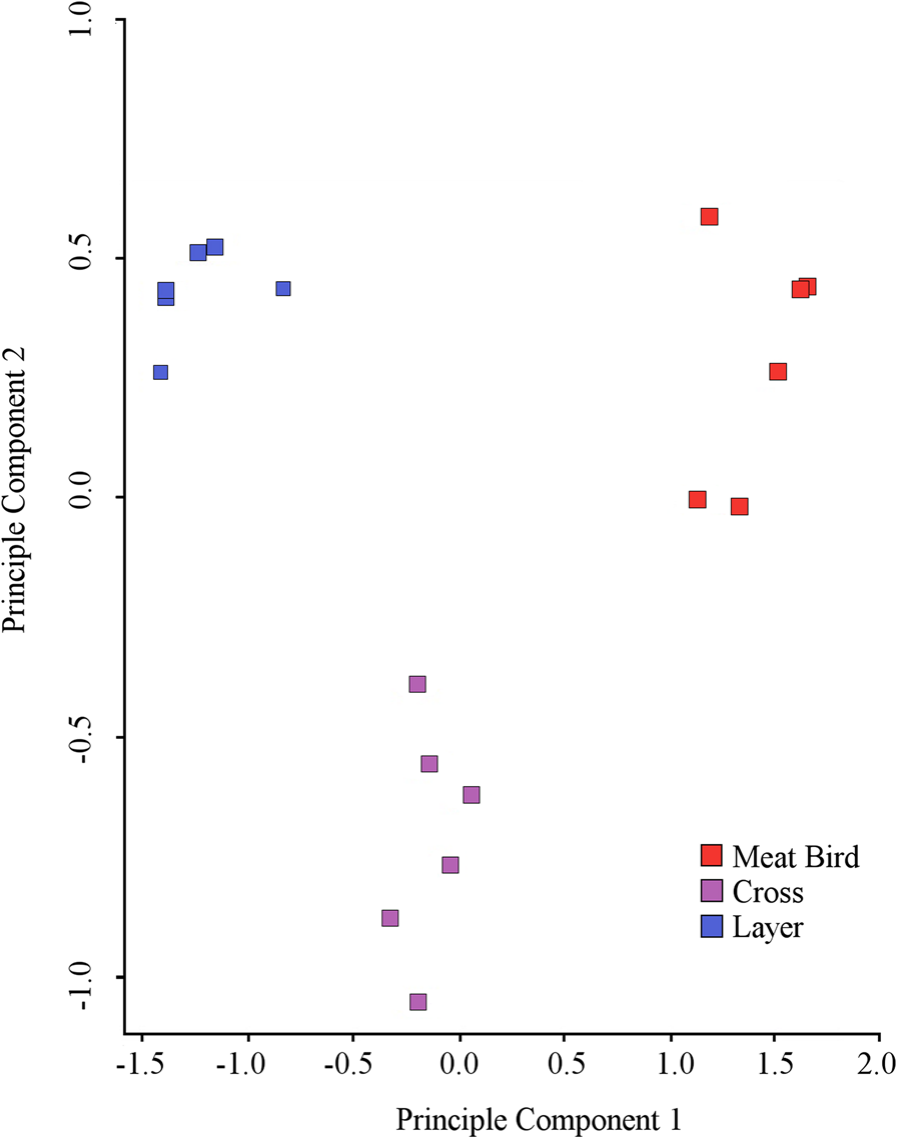
Principal component 1 vs principal component 2 analysis of meat bird (*n =* 6), cross (*n =* 6), and layer (*n =* 6) transcriptomes. Clusters can be seen for meat birds, layers and their F1 cross indicating gross transcriptome differences between the three groups of birds

RNA-Seq generated from 27,010,839 to 52,131,987 raw 2 × 100 paired end reads per sample with the average number being: meat bird (44,346,591), cross (40,568,610) and layer (35,862,746). After filtering the low quality reads, the average number of clean reads and percent retained were; meat bird (43,887,348; 99.0%), cross (40,146,845; 99.0%) and layer (35,447,280; 98.8%). Reads were mapped to the reference genome Galgal5.0. A total of 30,586 genes were identified among the chicken liver libraries, both known and novel. After removal of genes with no or low counts in all samples (< 1 CPM), 16,968 genes remained for analysis. Gross transcriptional analysis was undertaken using multidimensional scaling to determine how similar the transcriptomes were between the three groups. The results showed separate non-overlapping clusters of type; meat bird, layer and their F1 cross, indicating that each has a distinct transcriptome (Fig. 2).

### Identification of differential gene expression

**Fig. 3:**
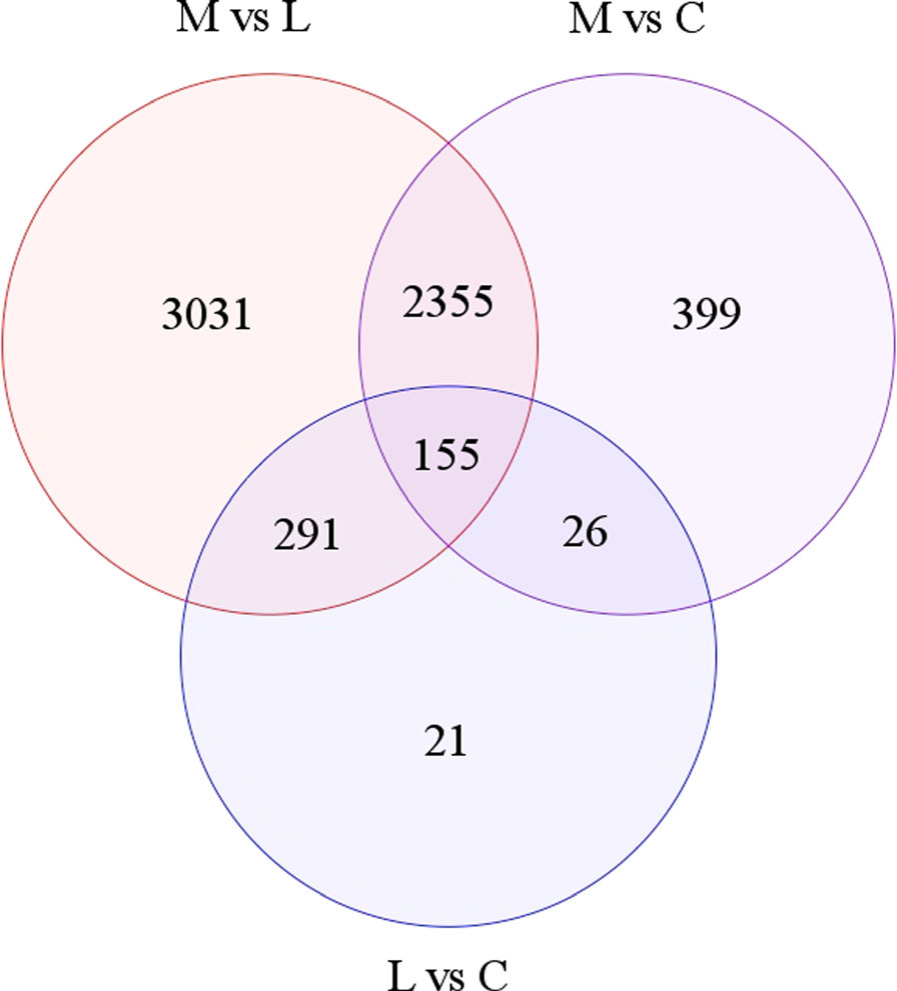
Venn diagram illustrating differential gene expression between meat birds (*n =* 6) vs layers (*n* = 6) (M v L); meat birds (*n* = 6) vs cross (*n* = 6) (M v C), and layers (*n =* 6) v cross (*n =* 6) (L v C)

Of the 16,968 genes expressed in at least 1 sample, 6278 genes were found to be DE for at least one of the comparisons of meat birds vs layers, meat birds vs crosses or layers vs crosses (Fig. 3). Of these 6278 genes identified as DE, 5832 were DE between meat birds and layers (19%), 2935 DE between meat birds and crosses (9.6%) and 493 DE between the layers and crosses (1.6%), highlighting that the transcriptome difference was greater in the meat birds than the layer or the cross. Percentages represent; the number of DE genes/ total genes (30,586), identified in the chicken libraries. Consideration of the transcriptome difference relative to body weight increases showed that a 1.8 fold increase in body weight from layer to cross was associated with a 1.6% transcriptome difference. The 2.0 fold increase in body weight between the cross and meat birds was associated with a 9.6% transcriptome difference, while the 3.5 fold bodyweight difference between meat birds and layers was associated with a 19% transcriptome difference. Comparisons between meat birds, crossed and layer birds identified 155 genes that are DE between all three groups. Of these 155 genes, 60% were found to be progressively upregulated in the direction meat bird > cross > layer, 38.1% down regulated meat bird < cross < layer, and 1.9% did not follow any directional pattern associated with growth rate.

### Characterisation of the 155 DE genes

The 155 DE genes were characterised in terms of abundance and fold change. Additionally, correlations were tested between the 155 DE genes with individual bodyweight. The top 10 most abundantly expressed genes were; alpha 2-HS glycoprotein (*AHSG*), fibrinogen alpha chain (*FGA*), fibrinogen gamma chain (*FGB*), fibrinogen beta chain (*FGG*), ferratin heavy polypeptide 1 (*FTH1*), compliment C4 (*C4*), acetyl-CoA transferase 2 (*ACAA2*), Dihydrolipoamide S-acetyltransferase (*DLAT*), saccharopine dehydrogenase (*SCCPDH*) and one unknown (NA) (Table 1). Of these top 10 most abundantly expressed, 6 were down regulated in meat birds (*AHSG, FGA, FGG, FGB, FTH1* and *C4*; meat bird < cross < layer), and 4 upregulated (NA, *ACCA2, DLAT* and *SCCPDH*; meat bird > cross > layer).

**Table 1:** The top 10 most abundantly expressed genes (mean ± SEM) presented as counts per million for male; meat (*n =* 6), cross (*n =* 6) and layer (*n =* 6) birds at d 14 post hatch

Gene ID	RefSeqID	Meat bird (*n* = 6)	Cross (*n* = 6)	Layer (*n* = 6)	Regulation ↑↓
*AHSG*	424,956	4433 ± 288	6103 ± 229	8049 ± 258	↓
*FGA*	396,307	5402 ± 166	6529 ± 142	7963 ± 332	↓
*FGB*	373,926	4041 ± 123	4834 ± 105	5976 ± 304	↓
*FGG*	395,837	3914 ± 99	4594 ± 72	5650 ± 259	↓
NA	NA	1603 ± 83	491 ± 163	7 ± 1	↑
*FTH1*	395,970	783 ± 20	912 ± 24	1129 ± 49	↓
*C4*	426,611	431 ± 25	558 ± 35	926 ± 55	↓
*ACAA2*	426,847	602 ± 32	473 ± 23	349 ± 11	↑
*DLAT*	419,796	590 ± 15	449 ± 8	327 ± 16	↑
*SCCPDH*	421,485	579 ± 31	470 ± 14	311 ± 11	↑

↓ Gene down regulated in meat birds (meat bird < cross < layer)

↑ Gene up regulated in meat birds (meat bird > cross > layer)

Fold changes were calculated using the mean CPMs for meat bird vs layer, meat bird vs cross and layer vs cross (Table 2). The largest fold change detected within the 155 DE genes was a 227-fold upregulation (CPM mean ± SEM) in meat birds (1602.90 ± 83.31) compared to layers (7.06 ± 0.89) for an uncharacterised gene (Un_24875). The second highest fold change was a 147.7 fold upregulation between the meat birds (1.12 ± 0.38) compared to layers (0.01 ± 0.01) for bacterial/permeability-increasing protein-like 3 (*BPIL3*). One individual bird with significantly increased expression in the meat bird group influenced the magnitude of the BPIL3 fold change. Six of the top 10 highest fold change (all genes) were novel and uncharacterised, highlighting gaps within the chicken genome. Among the top 10 characterised genes were *BPIL3*, LOC107055086 and LOC107057467 genes which have both been characterised as sperm-associated antigen 4 protein-like, Histamine N-methyltransferase-like (LOC771456), cyclin dependant kinase inhibitor 2B (*CDKN2B*), platelet glycoprotein VI-like (LOC10087809, leucine protein zipper 2 (*LUZP2*), butyrophilin subfamily 3 member A2-like (LOC107049070), ubiquitin C-terminal hydrolase L1 (*UCHL1*) and prostaglandin D2 synthase (*PTGDS*) (see Table 2).

**Table 2:** Top 10 fold changes of the 155 DE genes between meat birds, crossed and layer birds

Gene name	Gene description	RefSeqID	Mean CPM (±SEM)			Direction^a^	Fold Change^b^		
			Meat bird (*n* = 6)	Cross (*n* = 6)	Layer (*n =* 6)		M&L	M&C	L&C
Top 10 genes (all genes)									
NA	Uncharacterised	NA	1602.9 ± 83.3	491.3 ± 163.2	7.1 ± 0.9	↑	227.1	3.3	69.6
*BPIL3*	Bactericidal/permeability-increasing protein-like 3	419,290	1.1 ± 0.4	0.3 ± 0.1	0 ± 0	↑	144.9	4.4	32.8
NA	Uncharacterised	NA	0 ± 0	0.3 ± 0.1	1.8 ± 0.3	↓	121.6	20.1	6.1
LOC107055086	Sperm-associated antigen 4 protein like	107,055,086	0 ± 0	0.3 ± 0.1	1.4 ± 0.4	↓	99.3	19.8	5
NA	Uncharacterised	NA	2 ± 0.5	0.4 ± 0.1	0 ± 0	↑	57.4	4.6	12.5
NA	Uncharacterised	NA	12.8 ± 4.1	1.7 ± 0.5	0.2 ± 0.1	↑	56.4	7.5	7.5
LOC107057467	Sperm-associated antigen 4 protein like	107,057,467	0.1 ± 0	0.7 ± 0.2	3 ± 0.5	↓	49	11.4	4.3
LOC771456	Histamine N-methyltransferase-like	771,456	7.6 ± 1.3	1.1 ± 0.2	0.2 ± 0.1	↑	45.3	7.1	6.4
NA	Uncharacterised	NA	0 ± 0	0.5 ± 0.1	1.9 ± 0.4	↓	44.5	11.1	4
NA	Uncharacterised	NA	0.2 ± 0.1	1.7 ± 0.5	9.7 ± 2.2	↓	40.4	7	5.8
Top 10 characterised genes									
*BPIL3*	Bactericidal/permeability-increasing protein-like 3	419,290	1.1 ± 0.4	0.3 ± 0.1	0 ± 0	↑	144.9	4.4	32.8
LOC107055086	Sperm-associated antigen 4 protein-like	107,055,086	0 ± 0	0.3 ± 0.1	1.4 ± 0.4	↓	99.3	19.8	5
LOC107057467	Sperm-associated antigen 4 protein-like	107,057,467	0.1 ± 0	0.7 ± 0.2	3 ± 0.5	↓	49	11.4	4.3
LOC771456	Histamine N-methyltransferase-like	771,456	7.6 ± 1.3	1.1 ± 0.2	0.2 ± 0.1	↑	45.3	7.1	6.4
*CDKN2B*	Cyclin dependent kinase inhibitor 2B	395,076	363.8 ± 55.8	115.7 ± 17.6	12.9 ± 3.6	↑	28.2	3.1	9
LOC100857809	Platelet glycoprotein VI-like	100,857,809	0.3 ± 0.1	2.9 ± 0.6	7.6 ± 1.4	↓	27.5	10.3	2.7
*LUZP2*	Leucine protein zipper 2	423,001	0.9 ± 0.2	0.3 ± 0.1	0 ± 0	↓	26.7	3.5	7.5
LOC107049070	Butyrophilin subfamily 3 member A2-like	107,049,070	7.8 ± 0.9	2.3 ± 0.5	0.5 ± 0.1	↑	15.4	3.4	4.6
*UCHL1*	Ubiquitin C-terminal hydrolase L1	770,302	77 ± 5.9	16.9 ± 3.9	5.1 ± 0.8	↑	15.2	4.6	3.3
*PTGDS*	Prostaglandin D2 synthase	374,110	7.3 ± 1	21.8 ± 4.3	104.2 ± 26	↓	14.3	3	4.8

^a^Direction of regulation: ↑ Meat bird upregulated (meat bird > cross > layer); ↓ Meat bird downregulated (meat bird < cross < layer)

^b^Fold change comparisons: M&L = Meat bird and Layer; M&C = Meat bird and Cross; L&C = Layer and Cross

The 155 DE genes were correlated with individual bodyweight. Of the top ten correlated (Table 3), the highest correlation was between dihydrolipoamide S-acetyltransferase (*DLAT*), which is the E2 component on the pyruvate dehydrogenase complex, linking glycolysis to the citric acid cycle. DLAT was also among the top 10 most abundant of the 155 DE genes. Three of the top 10 genes correlated with bodyweight are novel and uncharacterised, e.g. LOC770248 and two unknown. Other genes highly correlated with bodyweight included quiescin Q6 sulfhydryl oxidase 1 (*QSOX1*), receptor accessory protein 5 (*REEP5*), myosin VI (*MYO6*), transmembrane protein 246 (*TMEM246*), cyclin G2 (*CCNG2*) and WW domain binding protein (*WBP2*).

**Table 3:** Top 10 genes with highest correlation with individual bodyweight

Chromosome	Gene ID	Gene Name	r^a^
24	*DLAT*	Dihydrolipoamide S-acetyltransferase	.968^**^
1	NA	N/A (Uncharactersied)	.956^**^
8	*QSOX1*	Quiescin Q6 sulfhydryl oxidase 1	.954^**^
Z	*REEP5*	Receptor accessory protein 5	.948^**^
3	*MYO6*	Myosin VI	.947^**^
1	NA3	N/A (Uncharactersied)	.947^**^
Z	*TMEM246*	Transmembrane protein 246	.946^**^
4	*CCNG2*	Cyclin G2	.945^**^
18	*WBP2*	WW domain binding protein	.944^**^
1	LOC770248	Uncharacterised	.943^**^

^**^Sig at *P* < 0.01

^a^Pearsons correlation coefficient

### Functional analysis of DE genes

All 6278 DE genes were analysed for GO terms and KEGG pathways using both edgeR and the web based tools in DAVID [31, 32]. There were 38 biological GO terms (GO: BP) identified for 5832 DE genes (*P* < 0.05) between meat bird and layer groups, 28 GO terms for 2935 DE genes between meat bird and the cross, and 19 GO terms for 493 DE genes between the layer and cross groups.

To understand the biological differences contributing to growth between the two strains and the F1 cross, we focused on the 155 DE genes among the meat birds, crossed and layer birds. For these 155 DE genes, 27 GO terms were identified (Table 4). Many of the GO terms were found to be significant due to the expression levels of *FGA*, *FGB* and *FGG*, which were among the most abundantly expressed genes. These three genes dominated 20 of the 27 GO terms identified, ranging from fibrinolysis, blood clot formation, fibrin clot formation, plasminogen activation, positive regulation of exocytosis, response to calcium ion and platelet aggregation. However, despite their high abundance, these genes had lower correlations with bodyweight than other DE genes (mentioned above), although still significant at *P* < 0.01. Of the 155 DE genes, ranked in order of correlation strength with bodyweight, *FGA* was 89th (*r* = − 0.874), *FGG* was 111th (*r* = − 0.085) and *FGB* was 124th (*r* = − 0.836). GO BP terms not largely dominated by *FGA, FGB* and *FGG* included positive regulation of glucose import, cellular response to oxidative stress and regulation of cell death. GO CC terms included chromatin and extracellular exosome. The extracellular exosome GO CC term (GO: 0070062) included 21 genes, 6 of which are in the top 10 most abundant (*AHSG*, *FGA*, *FGB*, *FGG*, *FTH1* and *ACCA2*), 2 in the top 10 fold changes (*PTGDS* and *UCHL1*), and 3 in the top 10 correlated with individual bodyweight (*QSOX*, *REEP5* and *MYO6*).

**Table 4:** Gene Ontology (GO) terms for the 155 DE genes

GO ID	GO Function	*P*-value	Gene ID^a^
GO Term BP			
GO:0042730	Fibrinolysis	7.20E-05	*FGA*↓*, FGB*↓*, FGG*↓*, CPB2*↓
GO:0034116	Positive regulation of heterotypic cell-cell adhesion	2.20E-04	*FGA*↓*, FGB*↓*, FGG*↓
GO: 0072378	Blood coagulation, fibrin clot formation	2.20E-04	*FGB*↓*, FGG*↓*, FBLN*↓
GO: 2000352	Negative regulation of endothelial cell apoptotic process	3.90E-04	*NFE2L2*↓*, FGA*↓*, FGB*↓*, FGG*↓
GO: 0051258	Protein polymerization	7.30E-04	*FGA*↓*, FGB*↓*, FGG*↓
GO: 0031639	Plasminogen activation	1.10E-03	*FGA*↓*, FGB*↓*, FGG*↓
GO: 0090277	Positive regulation of peptide hormone secretion	2.00E-03	*FGA*↓*, FGB*↓*, FGG*↓
GO: 0045921	Positive regulation of exocytosis	3.20E-03	*FGA*↓*, FGB*↓*, FGG*↓
GO: 0046326	Positive regulation of glucose import	6.30E-03	*INSR*↓*, NFE2L2*↓*, SLC1A2*↑
GO: 1902042	Negative regulation of extrinsic apoptotic signalling pathway via death domain receptors	7.20E-03	*FGA*↓*, FGB*↓*, FGG*↓
GO:0045907	Positive regulation of vasoconstriction	8.20E-03	*FGA*↓*, FGB*↓*, FGG*↓
GO:0050714	Positive regulation of protein secretion	1.00E-02	*FGA*↓*, FGB*↓*, FGG*↓
GO:0070527	Platelet aggregation	1.70E-02	*FGA*↓*, FGB*↓*, FGG*↓
GO:0051592	Response to calcium ion	1.80E-02	*FGA*↓*, FGB*↓*, FGG*↓
GO:0034599	Cellular response to oxidative stress	2.30E-02	*PARP1*↑*, SLC25A24*↑*, NFE2L2*↓
GO:0043152	Induction of bacterial agglutination	2.60E-02	*FGA*↓*, FGB*↓
GO:0010941	Regulation of cell death	3.40E-02	*JUN*↑*, SLC25A24*↑
GO:0070374	Positive regulation of ERK1 and ERK2 cascade	3.60E-02	*FGA*↓*, FGB*↓*, FGG*↓*, JUN*↑
GO:0007160	Cell-matrix adhesion	4.70E-02	*FGA*↓*, FGB*↓*, FGG*↓
GO Term CC			
GO:0005577	Fibrinogen complex	1.30E-05	*FGA*↓*, FGB*↓*, FGG*↓
GO:0005938	Cell cortex	1.20E-03	*FAM110C*↑*, FGA, FGG, MYO6*↑
GO:0031091	Platelet alpha granule	3.30E-03	*FGA*↓*, FGB*↓*, FGG*↓
GO:0072562	Blood micro-particle	7.00E-03	*AHSG*↓*, FGA*↓*, FGB*↓*, FGG*↓
GO:0000785	Chromatin	8.90E-03	*FBXO18, MAU2, CCND2*↑*, NFE2L2*↓
GO:0070062	Extracellular exosome	2.30E-02	*ACAA2*↑*, AKR1A1*↑*, AHSG, ANXA13*↑*, CDHR2*↑*, CPB2*↓*, ECI1*↓*, FTH1*↓*, FGA*↓*, FGG*↓*, FGB*↓*, FBLNI*↓*, INSR*↓*, MRAS*↓*, MYO6*↑*, PFKL*↑*, PTGDS*↓*, QSOX1*↑*, REEP5*↑*, TSTA3*↑*, UCHL1*↑
Go Term MF			
GO:0005198	Structural molecule activity	3.50E-02	*FGA*↓, *FGB*↓, *FGG*↓, NES↑
GO:0050662	Coenzyme binding	4.40E-02	*GCLC*, *TSTA3*

^a^Direction of regulation: ↑ Meat bird upregulated (meat bird > cross > layer); ↓ Meat bird downregulated (meat bird < cross < layer)

KEGG analysis of the 5832 DE genes between meat birds and layers revealed 13 pathways significantly enriched (*P* < 0.05). Pathways included; metabolic pathway (singular KEGG term), PPAR signalling pathway, biosynthesis of antibiotics, FoxO signalling pathway, cell cycle, drug metabolism, peroxisome, steroid biosynthesis, nicotinate and nicotinamide metabolism, glycine, serine and threonine metabolism, pentose phosphate pathway, glutathione metabolism and fatty acid metabolism. KEGG analysis of meat birds vs cross (2935 DE genes) identified 15 significantly enriched pathways, 10 pathways overlapped with those significant for meat birds vs layers including; metabolic pathway, PPAR signalling pathway, biosynthesis of antibiotics, FoxO signalling pathway, cell cycle, peroxisome, steroid biosynthesis, glycine, serine and threonine metabolism and the pentose phosphate pathway. Three pathways were significantly enriched for the layers vs cross; metabolic pathway, folate biosynthesis and FoxO signalling pathway. The metabolic and FoxO signalling pathway were the only two common pathways between the three types of bird identified (*P* < 0.05).

KEGG pathway analysis of the 155 genes DE between all three types of birds identified two enriched pathways at *P* < 0.05 (Table 5). Three genes were enriched for fructose and mannose metabolism (*P* = 0.024); 6-phosphofructo-2-kinase/fructose-2,6-biphosphatase 2 (*PFKFB2*), phosphofructokinase liver (*PFKL*), tissue specific transplantation antigen P35B *(TSTA3*). Five genes were associated with the FoxO signalling pathway (*P =* 0.001); cell cycle regulators; cyclin D2 (*CCND2*), cyclin G2 (*CCNG2*), cyclin-dependent kinase inhibitor 1B (*CDKN1B*), cyclin-dependent kinase inhibitor 2B (*CDKN2B*) as well as insulin receptor (*INSR*). Just falling out of significance at *P =* 0.053 was the glycolysis/gluconeogenesis pathway, involving three genes: *PFKL* (overlapping with fructose/mannose metabolism), which is rate limiting in glycolysis, catalysing the transformation of fructose-6-phospate to fructose-1,6-diphosphate [33]; glutamate transporter (*SLC1A2*), which was upregulated in the direction of meat birds (5.02 ± 0.70) crosses (2.16 ± 0.18) and layers (0.89 ± 0.14); and alcohol dehydrogenase (*AKR1A1* or *NADP*^*+*^), which was upregulated in the direction of meat birds (278.63 ± 12.84), crosses (230.01 ± 7.51) and layers (179.24 ± 6.45). *INSR* occurs in the FoxO pathway, and *SLC1A2* also overlaps with the GO term, GO: 0046326, positive regulation of glucose import.

**Table 5:** Pathways and associated genes identified as enriched by KEGG of the 155 DE genes between meat birds, crossed and layer birds

	Mean CPM^a^ (± SEM)			Direction^c^	Fold Change^b^		
Gene name	Meat bird (*n = 6)*	Cross *(n = 6)*	Layer (*n* = 6)		M&L	M&C	C&L
FoxO signalling pathway							
*CCND2*	183 ± 13	126 ± 9	79 ± 3	↑	2.3	1.4	1.6
*CCNG2*	348 ± 16	193 ± 15	104 ± 16	↑	3.4	1.8	1.9
*CCKN1B*	52 ± 1	60 ± 2	73 ± 2	↓	1.4	1.1	1.2
*CDKN2B*	364 ± 56	116 ± 18	13 ± 4	↑	28.2	3.1	9.0
*INSR*	86 ± 1	106 ± 4	131 ± 4	↓	1.5	1.2	1.2
Fructose and mannose metabolism							
*PFKFB2*	25 ± 2	31 ± 1	39 ± 1	↓	1.6	1.2	1.3
*PFKL*	296 ± 18	235 ± 7	170 ± 13	↑	1.7	1.3	1.4
*TSTA3*	49 ± 4	31 ± 1	21 ± 2	↑	2.4	1.6	1.5

^a^Mean CPM gene expression values are ‘counts per million’ transcripts to normalise for varying depth of sequence among samples

^b^Fold change comparisons: M&L = Meat bird and Layer; M&C = Meat bird and Cross; L&C = Layer and Cross

^c^Direction of regulation: ↑ Meat bird upregulated (meat bird > cross > layer); ↓ Meat bird downregulated (meat bird < cross < layer)

## Discussion

Liver transcriptomes of males of meat birds, F1 layer x meat bird crosses and layer birds were compared to identify DE genes between all three groups. Selection of the groups were based on their fast, moderate and slow growth potential, respectively. Day 14 post hatch was selected as the primary sampling time due to the rapid increase in growth seen in meat birds from 2 to 3 wks of age compared to other strains. By sampling at this time point, it was hoped to capture transcriptional changes at the beginning of rapid growth phase to further understand the biological factors associated with the high growth rates seen in meat birds.

The results of this study revealed that selection for growth or egg laying is associated with altered transcriptomes between meat and layer birds. Bodyweight at d 14 post hatch was 1.8 fold higher for crosses vs layers, and also 2.0 fold higher for meat birds vs crosses (meat bird > cross). The difference in transcriptomes associated with birds of differing bodyweights was quite remarkable. Of the total genes analysed, 1.6% were DE between crosses and layers; 9.6% DE between meat birds and crosses; and 19% DE between meat birds and layers. The differences in gene expression observed between the meat birds, layers and their F1 cross are not all driving the increases in bird size, particularly given the confounding effect of the many metabolic disturbances modern meat birds exhibit. These include; excessive fat deposition [5, 6], increased skeletal defects [7], pulmonary hypertension, sudden death syndrome [8, 9] and altered immune function [10]. However, it is likely that the drivers of growth are represented in the DE genes, particularly those that differ between all three groups.

GO and KEGG analyses of DE genes for meat birds vs layers, meat birds vs crosses and layers vs crosses identified overlapping biological functions that were affected and may contribute to the differential growth between types of birds. Two affected KEGG pathways were identified between all three comparisons; metabolic pathway (singular KEGG term) and the FoxO signalling pathway. The Forkhead box O (FoxO) genes central to this pathway are a family of transcription factors that regulate gene expression related to cell cycle regulation, cell survival, and metabolism, including glucose and lipid metabolism [34]. KEGG analysis of the 155 genes DE between each types of birds again identified the FoxO signalling pathway, enriched at *P* < 0.05. The fructose and mannose metabolism pathway was also enriched, with an overlap of genes involved in glycolysis, as well as the GO term ‘positive regulation of glucose import’. Among the functions of the FoxO signalling pathway is maintenance of homeostasis, particularly in response to stress [34].

FoxOs have previously been identified as potential candidate genes for growth in chickens. A genome-wide association study using a reciprocal cross between White Recessive Rock (WRR) and Xinghua (XH) chickens, identified a 1.5 Mb region on chromosome 1 containing 5 SNPs, including a SNP 8.9 kb upstream of *FoxO1* for bodyweight at 22–24 d and 70 d post hatch [35]. *FoxO1* contained two SNPs in the intron region of the gene; however, these two SNPs were not significantly associated with growth traits. The authors questioned whether a regulatory mechanism was involved in the significant SNP effects associated with growth traits located up and downstream of *FoxO1*. The most significant SNP for average daily gain at d 42 was in a region containing gene LOC770248, which is uncharacterised. Comparatively, LOC770248 was amongst the top 10 genes correlated (*r* = 0.934) with individual d 14 bodyweight in the present study. The identification of LOC770248 as a potential regulator of growth traits suggests further investigation is warranted to characterise the function of the encoded protein. More recently, RNA-Seq of the breast muscle of WWR and XH chickens at 7 wks post hatch identified *FoxO3* as a candidate gene (supported by siRNA analysis and association analysis) for further investigation into breast muscle growth in the chicken [36]. The significant enrichment of the FoxO signalling pathway in all comparisons in the current study strongly supports the contribution of this pathway to the growth differences between meat birds, layers and their F1 cross.

Of the 155 DE genes identified between the three types of bird, five genes associated with the FoxO signalling pathway were upregulated (meat birds > crosses > layers). These were insulin receptor (*INSR*), as well as genes essential for cell cycle regulation, cyclins *CCND2*, *CCNG2* and cyclin-dependent kinase inhibitors *CDKN2B*. Down regulation of *CDKN1B* was seen in meat birds compared with crossed and layer birds (meat birds < crosses < layers). Cyclins, such as *CCND2*, activate cyclin-dependent protein kinases (CDKs) which form complexes to transition the cell from one cell cycle state to another [37], for example; activation of cyclin-D dependent kinases initiates progression of the cell cycle through the G1 phase [38]. *CCND2* binds to several types of CDKs, with the main partners *CDK4* and *CDK6* [37]. We did not find *CDK4* in this gene set (of the 30,586), however, found abundant levels of *CDK6*, although not DE expressed. CDKs are normally present in the cell in excess of their cyclin partner [37], which was the case at the RNA expression level of *CDK6*:*CCND2* for meat birds, crosses and layers, with ratios of 1.3, 3.0 and 100.3 respectively. Interestingly, we found high DE of *CDKN2B* between all three groups (meat birds > crosses > layers) which inhibits the activity of *CDK4* and *CDK6*. *CDKN2B* is known to weaken the binding of D-type cyclins and as well as interact with the catalytic domains of *CDK4* and *CDK6* as a potent inhibitor of kinase activity [39]. Meat birds were the only group that had higher levels of *CDKNB2* relative to either *CDK6* or *CCDN2*, and the ratios for *CDKNB2:CCND2* and *CDKNB:CDK6* decreased (meat birds > crosses > layers) in both instances. *CCKNB2* was also amongst the top genes categorized by fold difference, being 28 fold higher in meat birds compared with layers.

Cyclin *CCNG2* was also upregulated in meat birds compared to cross and layer birds. Unlike ‘conventional’ cyclins that promote cell cycle progression, *CCNG2* upregulation in murine B cells is associated with cell cycle arrest or apoptosis in response to inhibitory stimuli, and conversely, *CCND2* is down regulated during G1 phase growth arrest [40, 41]. There is limited information of *CCNG2* activity in birds or in the liver for comparison. One study however compared Arbor Acres meat birds divergently selected for lean and fat lines, and identified *CCNG2* with a 0.209 and 0.249 lean/fat fold change at 2 and 4 wks respectively in liver tissue, which is similar to the fold change we observed between layers/meat birds (0.296) [42]. These studies, together with *CCNG2* being amongst the top 10 DE genes correlated with bodyweight in the present study, supports the differences between meat birds, crosses and layers being a result of differential cell cycle progression between the three types of birds.

Here we report that the liver (as a percentage of total bodyweight) reaches maximum size at d 14 post hatch in meat birds compared with the crossed and layer birds, where the ratio between liver and body size is lower, and the relative liver weight maximum is reached earlier, at d 7 post hatch. By d 28 there was no difference in relative liver weight (~ 3%) between any of the groups. In many plants and animals, organ scaling is controlled at the level of cell number [43]. However, for meat birds, although liver weight continued to increase at the same rate as the cross and layer birds from d 14-d 28, the expression studied, combined with the higher deceleration in relative liver weight from d 14 onwards in meat birds suggests that either; a) the total cell cycle time is increased or b) there is a decrease in growth fraction. Growth fraction has been defined as the number of cells remaining in the cell cycle vs the total organ cell number [44]. Therefore a decrease in growth fraction is likely due to fewer dividing cells as more remain in the G_0_ cell phase [44]. This would be supported by increased expression of *CCGN2* and *CDKNB2*. Thus, increased growth in the meat birds compared to cross and layer birds, likely results from hypotrophy (increased cell size) rather than hypoplasia (increased cell number). Hypotrophy via cellular polyploidy in the liver is not uncommon, with polyploid cells appearing late in fetal development, coinciding with terminal differentiation [45]. Polyploidy is associated with rapid growth by facilitating an increase in cell volume without division, which may permit cells to be more metabolically active [43, 46]. Without histological analysis on hepatocytes, increased polyploidy is speculative, however, there is evidence in this study to suggest the meat birds are more metabolically active.

The insulin receptor (*INSR*) was down regulated in broilers compared with cross and layer birds and was amongst the 155 genes with DE between the three groups. *INSR* was also enriched to the FoxO signalling pathway. The *INSR* pathway is conserved from flies to humans, and is a key sensor of nutrient availability, playing an important role in the control of cellular proliferation, cellular size and response to nutrient availability [47, 48]. Insulin regulates not only glucose metabolism, but also lipid homeostasis by increasing lipogenesis in the case of nutrient excess. In hepatocytes, activation of FoxO promotes the expression of key gluconeogenetic and glycogenolytic enzymes in the fasted state, resulting in increased hepatic glucose production [49]. In the fed state, high insulin blocks FoxO activity through the PI3-kinase (*PI3K*)-Atk pathway [49]. Atk phosphorylates the FoxO protein, retaining it in the cytoplasm in its inactive state [48–50]. This would favour glucose uptake and glycolysis. Furthermore, FoxOs have been shown to directly regulate the insulin signalling response to nutrients in C2C12 lines [48]. Upregulated insulin mRNA levels were associated with dephosphorylation of *FoxO1*, conversely down regulated insulin mRNA levels were associated with phosphorylation of *FoxO1* [48]. As phosphorylation of *FoxO1* results in decreased activation of *FoxO1*, it would be anticipated that this direct effect would result in decrease gluconeogenesis and increased glucose uptake and glycolysis. A major limitation in this study is that without functional analysis of the FoxO genes themselves, we cannot determine their activation status.

The lower expression levels of *INSR* in meat birds compared with crosses and layers however, is consistent with increased levels of phosphofuctokinase (*PFKL*; upregulated meat birds > crosses > layers), glutamate transporter *SLC1A2*, and *AK1A1,* which would be expected with increased levels of glycolysis, particularly as *PFKL* is a rate limiting enzyme in glycolysis. *PFKL* catalyses the transformation of fructose-6-phospate to fructose-1,6-diphosphate [33]. Furthermore, the pyruvate dehydrogenase complex (PDC) links glycolysis to the citric acid cycle. Therefore it is significant that dihydrolipoamide S-acetyltransferase (*DLAT*) was in the top 10 most abundantly expressed genes, upregulated in meat birds, and showed the highest correlation with bodyweight (*r* = 0.968). *DLAT* is the E2 component of the PDC, catalysing the oxidative reaction of pyruvate (end product of glycolysis) to acetyl-CoA in the mitochondria. Interestingly, chickens have been shown not to accumulate pyruvate in the liver, so the increase in *DLAT* is also consistent with the conversion to, and utilisation of acetyl-CoA in the mitochondria as soon as pyruvate is formed [51].

## Conclusion

In this study, we used RNA-Seq to show that the transcriptomes of meat birds, layers (and the F1 cross between them) are highly divergent, particularly between meat and layer type birds. Metabolic pathway (singular KEGG term) and the FoxO signalling pathway were identified as significantly enriched in comparisons between the three types of birds, with trends between meat, crossed and layer birds. Functional analysis of the 155 genes DE between all three strains also identified enrichment of the FoxO signalling pathway, particularly genes related to cell cycle regulation and the insulin receptor. These data suggest that differences in cell cycle regulation and glucose metabolism are associated with differences in growth rate, and provide evidence that meat birds have a higher rate of glycolysis. Functional analysis of the chicken hepatic FoxO genes and associated pathway targets warrants further investigation to determine the role of this pathway in regulating the growth of meat birds.
